# Comparison of the Efficacy of Jilo Animation Approach versus Conventional Tell-Show-Do (TSD) Technique on Cooperation and Anxiety Levels of Children during Dental Practice: A Randomized Controlled Clinical Trials

**DOI:** 10.30476/dentjods.2020.81897.1001

**Published:** 2020-12

**Authors:** Rasoul Sahebalam, Reihane Rafieinezhad, Marzie Boskabad

**Affiliations:** 1 Dental Research Center, Mashhad University of Medical Sciences, Mashhad, Iran; 2 Student Research Committee, Mashhad University of Medical Sciences, Mashhad, Iran; 3 Dental Materials Research Center, Mashhad University of Medical Sciences, Mashhad, Iran

**Keywords:** Animated Modeling, Anxiety, Cooperation, Children, Dentistry

## Abstract

**Statement of the Problem:**

Modeling is one of the non-pharmacological approaches to manage anxiety behavior and encourage children’s cooperation in dentistry. This method is based on social learning theory in which the children learn the skills of overcoming anxiety and adapting their skills and behaviors during dental treatment.

**Purpose:**

The aim of this study was to evaluate the effect of an animated-movie modeling approach, named Jilo, on cooperation and anxiety of children in comparison with the conventional Tell-Show-Do (TSD) method in a dental setting.

**Materials and Method:**

In this randomized controlled trial, 50 healthy children (aged 4-6) with no history of dental treatment were recruited and randomly allocated to experimental (n= 24) and control (n= 24) groups. During the first visit, the experimental group watched the Jilo animated movie. After a 30-minute break, they received prophylaxis followed by fluoride therapy. The control group received the conventional TSD technique and then underwent prophylaxis and fluoride therapy. One week later, a dental restoration, which required mandibular nerve block anesthesia, was performed for both groups. The cooperation and anxiety levels of patients were evaluated during two visits using Venham Clinical Cooperation Scale (VCCS) and Venham Clinical Anxiety Scale (VCAS).

**Results:**

The mean VCAS in the experimental group was significantly lower compared to the control group during the first and second visits (*p*= 0.008 and *p*= 0.044, respectively). The mean VCCS was also significantly lower in the experimental group during the first (*p*= 0.015) and second visits (*p*= 0.019) compared to the control group.

**Conclusion:**

The application of animated-movie modeling (Jilo) can be recommended as an effective method for preparation of children before the dental treatment session.

## Introduction

Behavior management of children is often a major challenge that a dentist has to cope with during a treatment session. Fear and anxiety of children with regard to the prospect of dental treatments are serious problems that families and pediatric dentists need to address [ 1 - 2 ]. Unless it is cured, this problem might persist until adulthood [ 3 ]. As far as children are concerned, there is a strong correlation between the anxiety induced by dental treatment and positive prognosis in dentistry; therefore, anxiety management is of paramount importance as a significant factor, which contributes to success in dental treatments [ 4 ]. The state of anxiety experienced during a dental treatment is a common condition that, in most cases, seems to originate from childhood. Consequently, a child might refuse to cooperate and may show disruptive behavior during a treatment session. This could result in multiple complications, including: delayed treatment; occasional failure in conducting the treatment using conventional methods of behavior guidance; refusal to observe dental care; and, eventually, increased prevalence of dental caries [ 5 - 7 ]. The first visit to a dentist is very important in terms of shaping the child’s behavior, their attitude, and ensuring treatment success. Pediatric dentists often rely on the Tell-Show-Do (TSD) technique to manage a child’s anxiety during the examination session prior to the treatment visit. This technique involves explaining the work that is going to be performed on the patient before the actual treatment practice. Next, the child is exposed to a simulation of the work to be done. This technique is based on the premise of acquisition and is conducted by the dentist within the actual treatment environment [ 8 ].

Other prevalent behavior management methods include desensitization; visual modeling; play therapy; animated-movie modeling, and pharmacological behavior control [ 8 - 9 ]. According to the modeling technique, which was first introduced by Bandura in 1967, a child is expected to acquire and show a behavior consistent with that of a model they have previously been exposed to in a similar context [ 10 ]. A sibling of the patient is employed to educate the child about the expected behavior during the session prior to the treatment visit.

Several studies have focused on the efficacy of animated-movie modeling in reducing the dental treatment anxiety experienced by children. The results indicate that the application of animated-movie modeling can be just as effective as other approaches, such as live modeling techniques and various desensitization methods. Unlike techniques based on collective learning, the animated-movie modeling approach does not impose spending a great deal of time on the dentist and the caring team [ 8 ]. The present study was designed to evaluate the effect of modeling on the behavior of a sample population of Iranian children using an animated-movie, which simulated an actual dental-office environment with animated characters. Furthermore, the study aimed to compare the efficacy of this method versus the conventional TSD technique.

## Materials and Method

This randomized controlled single blinded parallel-group clinical trial was approved by the Research and Ethics Committee, Faculty of Dentistry, Mashhad University of Medical Science, Mashhad, Iran under the serial code: IR.mums.sd.REC.1395.139. This study was performed considering the consolidated standards of reporting trials statement for randomized trial. It was conducted and reported in accordance with the declaration of *Helsinki* for Biomedical Research Involving Human Patients. 

The participants were selected from eligible patients who referred to Department of Pediatric Dentistry, Faculty of Dentistry, Mashhad University of Medical Science from February to March 2017. Preparation of the patients and clinical procedures were conducted in the clinical Department of Pediatric Dentistry from March 2017 to June 2017.

A study population of children aged 4 to 6, who met the following inclusion and exclusion criteria, was selected with goal-oriented sampling strategy. The inclusion criteria depicted as (1) needing at least one restoration involving a mandibular molar and requiring inferior alveolar nerve block anesthesia, (2) having no record of admission in hospital, (3) having no previous experience of dental treatment, and (4) negative history of any particular underlying disease. The exclusion criteria described as (1) emergency needs such as drainage of abscess or extraction, (2) behavioral disorder or cognitive impairment, and (3) pulpal exposure of the tooth during caries removal. After a brief examination of the patients to verify their eligibility to enter the study, a pediatric dentist conducted an interview was with the children’s parents or their primary caregivers to obtain medical and dental history. Then the purpose and method of the study were described for the parents. The informed consent form and the personal information section of the patient’s file were signed and filled by parents.

The sample size in this study was considered 25 in each group regarding Kebriaee *et al*. study [ 11 ] with the type 1 error level of 5%, type 2 error level of 20%, and dropout rate of 10%. Eventually, 50 patients were entered into the study and randomly assigned to two equal groups of control (n=25) and experimental (n= 25). Two appointments were scheduled for each patient with one-week interval between. One patient from each group was excluded from the study after they refused to show up for the second treatment session.

### First appointment

Adopting a balanced randomized block design and usin-g the randomizer.com website, each patient was allocated to either of the following groups by the second author.

### The control group: Tell-Show-Do group (TSD)

In this group, the patient learned about the dental procedures and instruments using Tell-Show-Do technique during the first visit. Then patients received prophylaxis with rubber cup and low speed handpiece followed by fluoride therapy with %1.1 sodium fluoride gel as a preparation stage to familiarize these patients with the environment of a dental office. At the end of the session, a reward gift was given to the patient to serve as an encouraging positive reinforcement.

### The experimental group: Modeling using an animated movie (Jilo)

During the first visit, the patient and the accompanying parent were led to sit in a quiet room and watch an animated movie entitled “Jilo goes to a dentist” (https:// www.aparat.com/v/g7wFt). The storyline of this animation is set in the animal world; one day, a young bear called Jilo goes to a dentist with his mother. Dental instruments and procedures are designed in the form of objects and concepts that a child can understand, and that involve their imagination. For example, the suction procedure has been modeled using an elephant's trunk and its power to suck in water. After watching the animated movie, the patients were let to rest for 30 minutes, which was followed by prophylaxis and fluoride therapy in the same manner as the control group.

One week later, during the second visit, the patients were reminded of the atmosphere of the animated movie and its characters before undergoing a restoration that required mandibular nerve block injection.

### Second appointment

**Figure 1:**
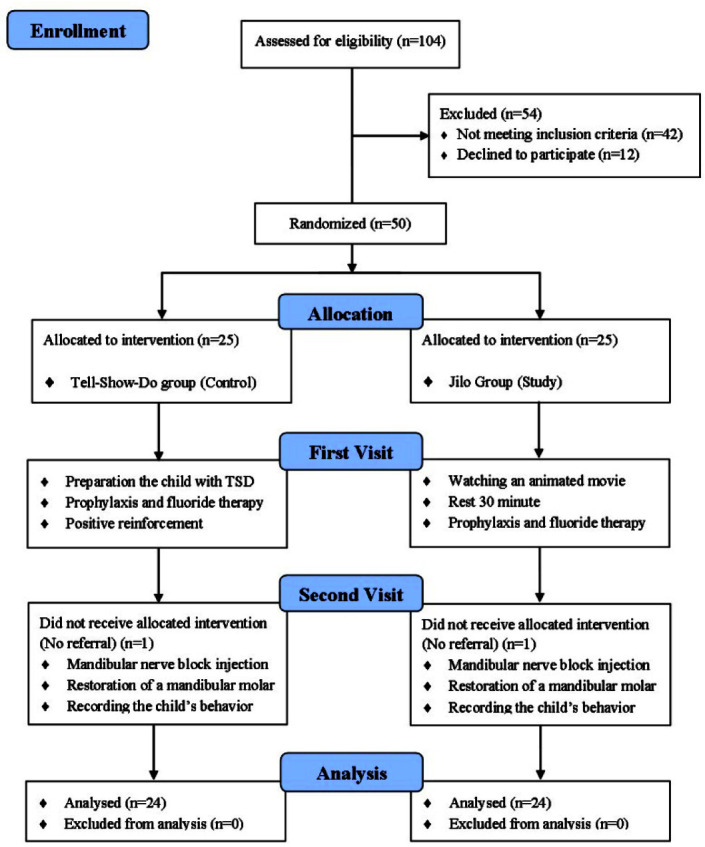
Flowchart of the study design

A week after the first appointment, the patient returned to the clinic. The patient was asked to take a seat on a dental unit without the presence of their parent. Mandibular nerve block injection was performed in conjunction with topical anesthesia. Once local anesthesia was ensured, an access cavity was prepared to remove the caries and restore the tooth. It was made sure that all patients were treated under similar circumstances, such as the performance of the dentist and their assistant, instruments and materials, treatment environment, treatment time (half an hour past breakfast in the morning) and duration of the treatment (about one hour). Similar trained practitioner (second author) did all the procedures for both groups (Figure 1).

### Behavioral evaluation

In all stages in the first and second visit, a camera (Panasonic, NV-GS35GC) was fixed in position- directed at the patient- filming their face, hands and feet, in order to accurate recording of the patient’s behavior.

Two evaluators, who were blind to the experimented techniques, watched the recorded films of both groups separately. They assessed the behavior of all patients (cooperation and anxiety levels) during all stages of prophylaxis, fluoride therapy, anesthetic injection, and dental instrumentation with a handpiece considering the recorded videos. This assessment was performed by using Venham Clinical Cooperation Scale (VCCS) and Venham Clinical Anxiety Scale (VCAS), which are among the most frequent behavior scoring models [ 12 - 13 ]. These scales contain six defined behavioral levels that range from 0 to 5. The highest score defines the highest anxiety level, or the least cooperation (Table 1 and 2).

**Table 1:** Venham Clinical Cooperation Scale (VCCS) description [14]

Score	Definition
1	**Uneasy:** concerned, may protest briefly to show discomfort, hands remain down or partially raised. Tense facial expression. Capable of cooperating
2	**Tense:** tone of voice, questions, and answers reflect anxiety. During stressful procedure, verbal protest, crying, hands raised with tension, but not interfering very much. Protest more distracting and troublesome. Child still complies with request to cooperate.
3	**Reluctant:** prominent verbal protest, crying. Using hands to try to stop procedure. Treatment proceeds with difficulty.
4	**Interference:** general crying, body movements sometimes needing physical restraint. Protest disrupts Procedure.
5	**Out of contact:** hard loud swearing, screaming unable to listen, trying to escape. Physical restraint required.

**Table 2:** Venham Clinical Anxiety Scale (VCAS) description [14]

Score	Definition
1	Mild, soft verbal protest or (quiet) crying as a signal of discomfort, but does not obstruct progress. Appropriate behavior for procedure, i.e., slight start at injection, “ow” during drilling if hurting, etc.
2	Protest more prominent. Both crying and hand signals. May move head around making it hard to administer treatment. Protest more distracting and troublesome. However, child still complies with request to cooperate.
3	Protest presents real problem to dentist. Complies with demands reluctantly, requiring extra effort by dentist. Body movement.
4	Protest disrupts procedure, requires that all of the dentist’s attention be directed toward the child’s behavior. Compliance eventually achieved after considerable effort by dentist, but without actual physical restraint. May require holding child’s hands or other parts of the body to start treatment. More prominent body movement.
5	General protest, no compliance, or cooperation. Physical restraint is required.

If there were any differences in the scores of the reviewers, the film was reviewed and both reviewers achieved an agreement on a score.

Thus for each patient, two scores on the levels of anxiety and cooperation for the first visit (prophylaxis and fluoride therapy) and two scores for the second visit (restoration), were obtained. In order to confirm the intra-examiner reliability, 5 patients from each group were randomly selected and their films were re-evaluated and scored, and correlation coefficient was obtained. No changes were made to the design and outcomes of the study after trial commenced.

### Statistical analysis

Data were analyzed with SPSS version 21. Normal distribution of data was determined using Shapiro-Wilk Normality test. Mann-Whitney U test was used to compare VCCS and VCAS between two study groups. In addition, the relationship between the variables was assessed using Spearmen Correlation Coefficient. The significant level was considered as *p*< 0.05.

## Results

In this study, 48 patients participated in both visits including 17 boys (35.4%) and 31 girls (64.6%) with a mean age of 5.32±6.33 years. The patients were divided into control and Jilo (experimental) groups (n= 24 in each group). Table 3 shows demographical information of participants in each group. There was no significant difference in gender distribution (*p*= 0.113) and mean age between the two groups (*p*= 0.503). The correlation between the two evaluators showed that there was a high agreement between the two evaluators in assessing the level of anxiety and cooperation. Shapiro Wilk's test revealed non-normal distribution of the data.

**Table 3:** Demographic information of the studied patients of two groups and comparison between the groups

Groups	N	Sex		Age
		Male	Female	
Experimental Group (Jilo)	24	6(25%)	18(75%)	5.16±0.61
Control Group (TSD)	24	11(45.8%)	13(54.2%)	5.29±0.65
Total	48	17(35.4%)	31(64.6%)	5.23 0.63
*p* Value		0.131		0.503

Comparison between the two groups was done using Chi square test for sex and T-Test for age.

**Figure 2:**
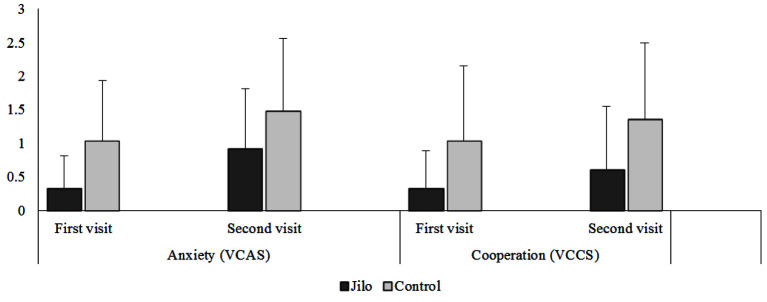
Mean±SD of the VCAS (Venham Clinical Anxiety Scale) and VCCS (Venham Clinical Cooperation Scale) in two visits of two groups

**Figure 3:**
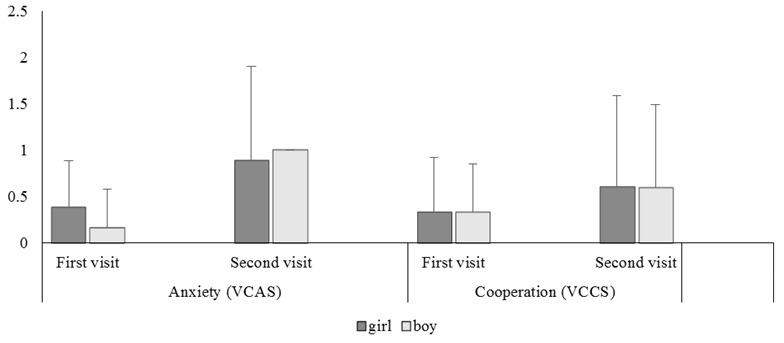
Mean±SD of the VCAS (Venham Clinical Anxiety Scale) and VCCS (Venham Clinical Cooperation Scale) in two visits of Jilo group

**Figure 4:**
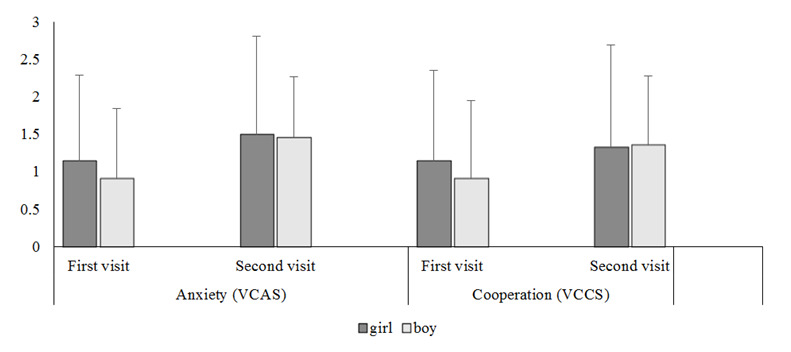
Mean±SD of the VCAS (Venham Clinical Anxiety Scale) and VCCS (Venham Clinical Cooperation Scale) in two visits of control group

The results showed that patients in the control group exhibited significantly higher levels of anxiety and non-cooperation than the Jilo group, during both the first and second sessions. In the first visit, the mean VCAS and VCCS scores of the control group were 1.04±1.04 and 1.04±1.12, respectively, versus 0.33±0.48 and 0.33±0.56 in the Jilo group (*p*= 0.008 and *p*= 0.015 respectively). In the second visit, the control group achieved mean VCAS and VCCS scores of 1.48±1.08 and 1.35± 1.15, respectively, versus 0.91±0.90 and 0.61±0.94 in the Jilo group (*p*= 0.044 and *p*= 0.019 respectively) (Figure 2). Considering the effect of gender on the level of anxiety and cooperation, there were no statistically significant differences between two genders in both groups (Figure 3 and 4).

Moreover, in assessing the effect of age, it was found that the age of participants did not have a significant effect on the level of anxiety and cooperation between the two groups in two visits (Table 4).

**Table 4:** Spearman correlation coefficient of the age with VCAS (Venham Clinical Anxiety Scale) and VCCS (Venham Clinical Cooperation Scale) in two visits and two groups

Group		Anxiety (VCAS)		Cooperation (VCCS)	
		First visit	Second visit	First visit	Second visit
Experimental (Jilo)	Correlation	0.039	-0.3	-0.05	0.06
	*p* Value	0.85	0.15	0.79	0.76
Control (TSD)	Correlation	-0.34	-0.04	-0.21	0.017
	*p* Value	0.09	0.83	0.31	0.93

The comparison of the mean changes in anxiety and cooperation in two visits between the two groups showed that in both groups, the level of anxiety and cooperation in the second visit were lower than the first visit and in the Jilo group, this decrease was more pronounced. However, there was no significant difference in the reduction of anxiety and cooperation in the second visit between the two groups (Table 5).

**Table 5:** Comparison of Mean±SD of changes of VCAS (Venham Clinical Anxiety Scale) and VCCS (Venham Clinical Cooperation Scale) in two visits between two groups

Groups	Anxiety (VCAS)	Cooperation (VCCS)
Experimental (Jilo)	-0.56±0.72	-0.3±0.97
Control (TSD)	-0.39±0.97	-0.26±1.01
*p* Value	0.489	0.88

Comparison between the two groups was done using Mann- Whitney U test

## Discussion

The modeling technique is based on a psychological theory according to which people learned how to behave in a particular situation through observing others’ behavior therein. This technique involves employing a model, which sets an appropriate example of behavior. The child is then expected to show the required correct behavior, which is rewarded by an encouraging response and reinforced through a positive result.

The desirable outcome will be reduced anxiety of the child [ 15 ]. The Tell-Show-Do (TSD) technique is commonly used to acquaint the patient with the unfamiliar environment of a dental office as well as the treatment procedures, particularly at onset. In 1959, Addlestone introduced this technique, which rests on a number of tenets from the acquisition/learning theory, and, ever since, it has served as the foundation for child behavior control [ 8 ]. 

This technique can be applied as a standardized approach. Most often, pediatric dentists utilize imaginary expressions and substitute vocabulary in order to enhance the clarity of messages they need to get across as well as to improve the conceptualization of the child. This substitute language resembles a second language whereby the pediatric dentist and the child communicate [ 10 ].

The present study was designed to evaluate the efficacy of watching an animated movie, which educates the child about the entire dental-office environment and the treatment procedures by involving the child’s imagination. The produced animation simulates dental instruments and procedures using objects and animated animals that a child can readily conceptualize.

The findings of this study suggest that, when the child comes to a dental treatment office, watching an animation with animated characters, which engages the child’s imagination, can be more effective in reducing the child’s anxiety and improving their cooperation than the TSD technique alone.

The target group of this study was defined as 4-6-year-old children, similar to another study by Paryab *et al*. [ 16 ] and many others [ 16 - 17 , 19 ]. The study population was selected from children at preschool stage to eliminate the confounding influence of school on the behavior of the child as well as their cognitive level [ 20 ], and to remove other age-related confounding factors. As a constant finding, in most studies, behavioral management problems are more common in early childhood, especially in preschool children [ 9 , 21 ].

In this study, the two groups did not have a significant difference in age and sex, and therefore there was no confounding factor in this view. The mean of anxiety and cooperation in the first and second visits was not significant in the Jilo group and in the control group between boys and girls. As a result, there was no significant relationship between the gender and the level of anxiety and child cooperation similar to previous studies [ 20 , 22 - 23 ]. The patients participating in the present study had no prior experience of a dental visit, and, therefore, they experienced visiting a dental office for the first time. Since a traumatic dental experience in the past can result in increased levels of fear and anxiety in children with respect to further treatment sessions- and possibly lead to negative, disruptive behavior during treatment, this confounding factor was removed from the study. Children with pain and emergency problems were excluded because the pain experience can increase children’s anxiety and influence their cooperation with the dentist.

Forty-eight children, in two groups of 24 patients each, were included in the current study. In a similar study, Paryab *et al*. considered a sample population size of 46 children, which is highly consistent with the present study. However, the sample size of the present study was greater than the sample size in a study performed by Salem *et al*. and Melamed *et al*. [ 18 , 24 ].

In the Jilo group, the level of anxiety slightly increased and the level of cooperation showed a slight decrease during the second visit, but these changes were not statistically significant with the changes in the control group. Therefore, it is fair to claim that exposure to the animated movie used in this study played an effective role in reducing anxiety and improving cooperation of the patients. In addition, the one-week interval between the first visit, when the animation was watched, and the second visit indicated the long-term effect of the experimental technique at least for one week.

To date, various tools have been introduced for the purpose of evaluating and scoring the cooperation and anxiety levels in children with regard to dental visits [ 25 ]. Similar to the method used by Paryab *et al*. [ 16 ], this experiment utilized Venham index for scoring the patients’ behavior. Accordingly, the cooperation and anxiety levels of the children, observed in the recorded films, were measured using VCCS and VCAS, which are regarded as common behavior scoring models. These models (Venham index) have been generally confirmed in terms of reliability and validity, and have been widely used in dental research to assess children’s anxiety and negative responses [ 13 ].

It is worth mentioning that the application of Behavior index alone may not be sufficient when it comes to evaluation of the anxiety level. Thus, it is recommended to utilize a combination of behavior index and physiological index in future studies.

## Conclusion

The findings of the current study reveal that the animated-movie modeling technique can be used to produce a desirable effective influence during the preparation prior to visit as well as the dental treatment sessions involving children aged 4-6. This technique can be used in conjunction with the conventional Tell-Show-Do technique to generate a positive synergic effect.
